# Unilateral proptosis as the primary presentation of Stage IVb Juvenile Nasopharyngeal Angiofibroma with direct external carotid artery supply: Case report

**DOI:** 10.1016/j.radcr.2026.03.024

**Published:** 2026-04-16

**Authors:** Lama Kayyali, Ammar Alshikh Ali, Alae Kayyali

**Affiliations:** Faculty of Medicine, University of Aleppo, University Square, Aleppo, Syria

**Keywords:** Juvenile Nasopharyngeal Angiofibroma, Unilateral proptosis, Holman–Miller sign, External carotid artery, Case report

## Abstract

Juvenile Nasopharyngeal Angiofibroma (JNA) is a benign, hypervascular tumor that typically presents with nasal symptoms. However, when it presents with isolated ophthalmic features, it can lead to significant diagnostic pitfalls. We report a rare “ophthalmic-first” presentation of a Stage IVb JNA in a 19-year-old male. The patient presented only with progressive unilateral proptosis for 7 months, initially misdiagnosed as a traumatic carotid-cavernous fistula due to a history of minor head trauma. Advanced imaging (computed tomography and magnetic resonance imaging) corrected the diagnosis by identifying a 72 × 49 mm nasopharyngeal mass with a positive Holman–Miller sign and characteristic “flow voids”. Unique to this case, computed tomography Angiography revealed a direct arterial feeder originating from the external carotid artery rather than the typical maxillary artery. Based on Fisch Stage IVb classification, the tumor demonstrated extensive local aggression, invading the orbit and compressing the left temporal lobe. This case highlights a diagnostic challenge where JNA presented with an atypical “ophthalmic-first” manifestation, mimicking a carotid-cavernous fistula, because the patient only complained of proptosis and overlooked his nasal symptoms. Radiologically, the tumor was advanced (Fisch Stage IVb) and exhibited a unique vascular pattern with a direct feeding branch from the external carotid artery, significantly increasing the risk of massive hemorrhage. Due to these multidisciplinary management challenges, the patient required specialized preoperative embolization and vascular control. However, the case highlights a common clinical hurdle, as the patient was lost to follow-up before intervention. This highlights the need for high clinical suspicion and a centralized tracking system for managing such complex, high-risk vascular tumors. JNA must be considered in the differential diagnosis of adolescent males presenting with unilateral proptosis, even when classic symptoms like epistaxis are mild or initially underreported by the patient. Identifying atypical vascular patterns, such as direct feeders from the external carotid artery, is essential for meticulous surgical planning and effective hemorrhage control in advanced Stage IVb cases.

## Introduction

Juvenile Nasopharyngeal Angiofibroma (JNA) is a benign, locally aggressive fibrovascular tumor. It arises primarily within the nasopharynx or the posterior nasal cavity, predominantly affecting adolescent males. Clinical presentation typically includes unilateral nasal obstruction and recurrent epistaxis. In advanced cases, the tumor may lead to secondary symptoms such as facial swelling, headaches, anosmia, or cranial neuropathies. Because this tumor is rich in blood vessels and can spread to nearby tissues, early diagnosis is essential to [[1], [2], [3]] prevent serious complications.

Although its exact site of origin remains controversial, it is widely believed to arise from the superior lip of the sphenopalatine foramen. JNAs exhibit a slow growth pattern, initially expanding within the nasopharynx and later spreading to the pterygomaxillary space; over time, these tumors can erode bone and invade critical structures such as the infratemporal fossa, the orbit, and the middle cranial fossa [4].

To guide surgical planning and assess the extent of invasion, several staging systems [5,6] are utilized, most notably the Fisch and Radkowski classifications.

In this report, we primarily adopt the Fisch staging system to categorize the tumor based on its involvement of the infratemporal fossa, orbit, and intracranial compartments.

**Fig. 1:**
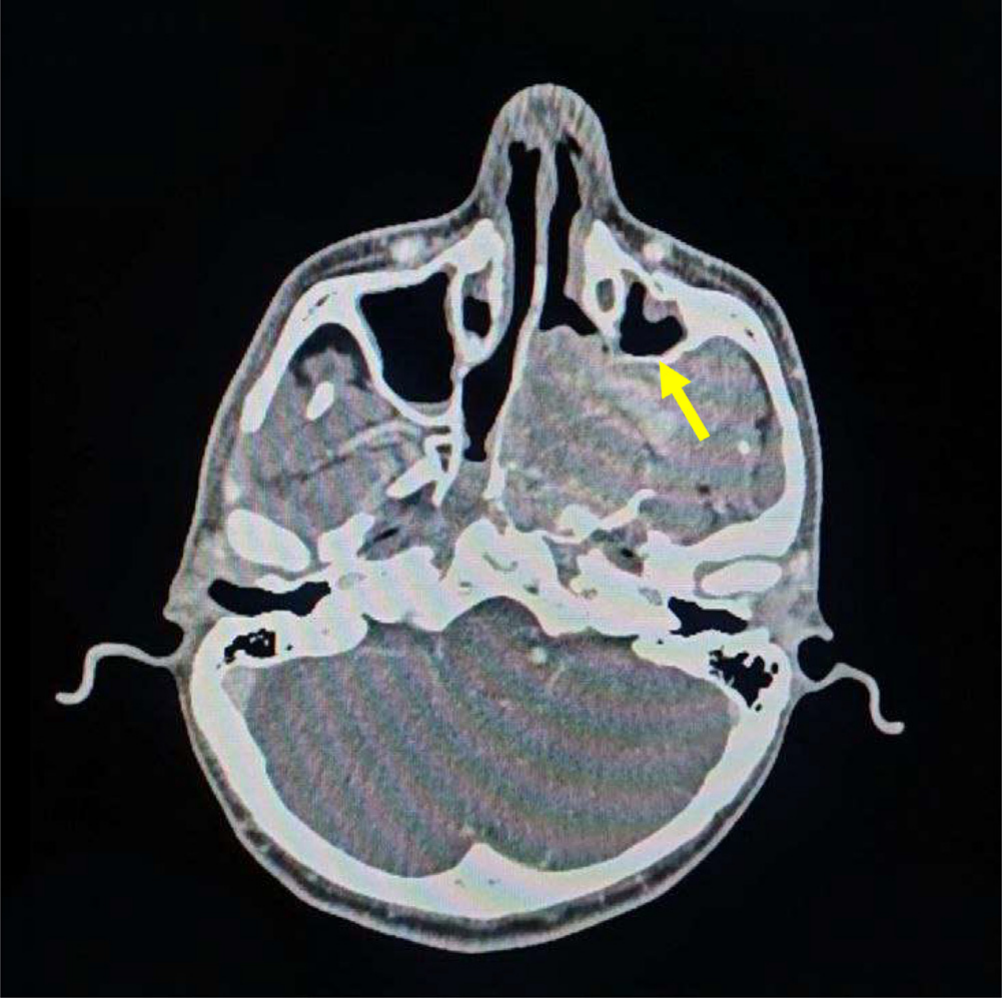
Axial noncontrast CT scan showing the pathognomonic Holman–Miller sign (antral sign), characterized by the anterior bowing of the posterior wall of the left maxillary sinus (arrow) due to the mass effect from the nasopharyngeal tumor.

Clinically, the most common symptoms are painless nasal obstruction and epistaxis. However, due to the tumor’s extreme vascularity, incisional biopsy is contraindicated, and diagnosis depends on multiplanar imaging like computed tomography (CT) and magnetic resonance imaging (MRI). A pathognomonic radiological feature is the Holman–Miller sign (antral sign) (Fig. 1), characterized by the anterior bowing of the posterior wall of the [4] maxillary antrum.

While nasal symptoms are the hallmark of JNA, atypical presentations can occur depending on the tumor’s extent. The presence of proptosis serves as a crucial clinical marker for orbital extension, typically representing advanced stages of the disease [4] (Stage IV).

In this report, we present a rare case of a 19-year-old male with Stage IVb JNA, where unilateral proptosis was the primary and only initial manifestation.

We detail the diagnostic and therapeutic challenges of managing this complex presentation [7] while adhering to CARE 2013 guidelines for clinical case reporting.

## Case presentation

A 19-year-old male, with no significant prior medical or surgical history, presented to the ophthalmology department. He complained of progressive, painless proptosis and swelling of the left eye that had persisted for 7 months. Interestingly, the patient associated the onset of these symptoms with a prior history of minor head trauma, which led to an initial clinical suspicion of a traumatic vascular abnormality.

Upon initial ophthalmic evaluation, an ocular ultrasound was performed, raising suspicion of a carotid-cavernous fistula (CCF) due to the traumatic history. However, the patient maintained normal visual acuity, and his extraocular movements were intact. A month and a half later, the patient was referred for advanced radiological imaging. Upon more detailed history taking during this period, the patient revealed a long-standing history of mild, intermittent epistaxis and nasal obstruction, which he had not initially reported as he did not perceive them to be related to his ocular condition. Preoperative laboratory investigations showed a Hemoglobin level of 13.1 g/dL, a prothrombin time of 14 seconds, and a Prothrombin Activity of 100%.

**Fig. 2:**
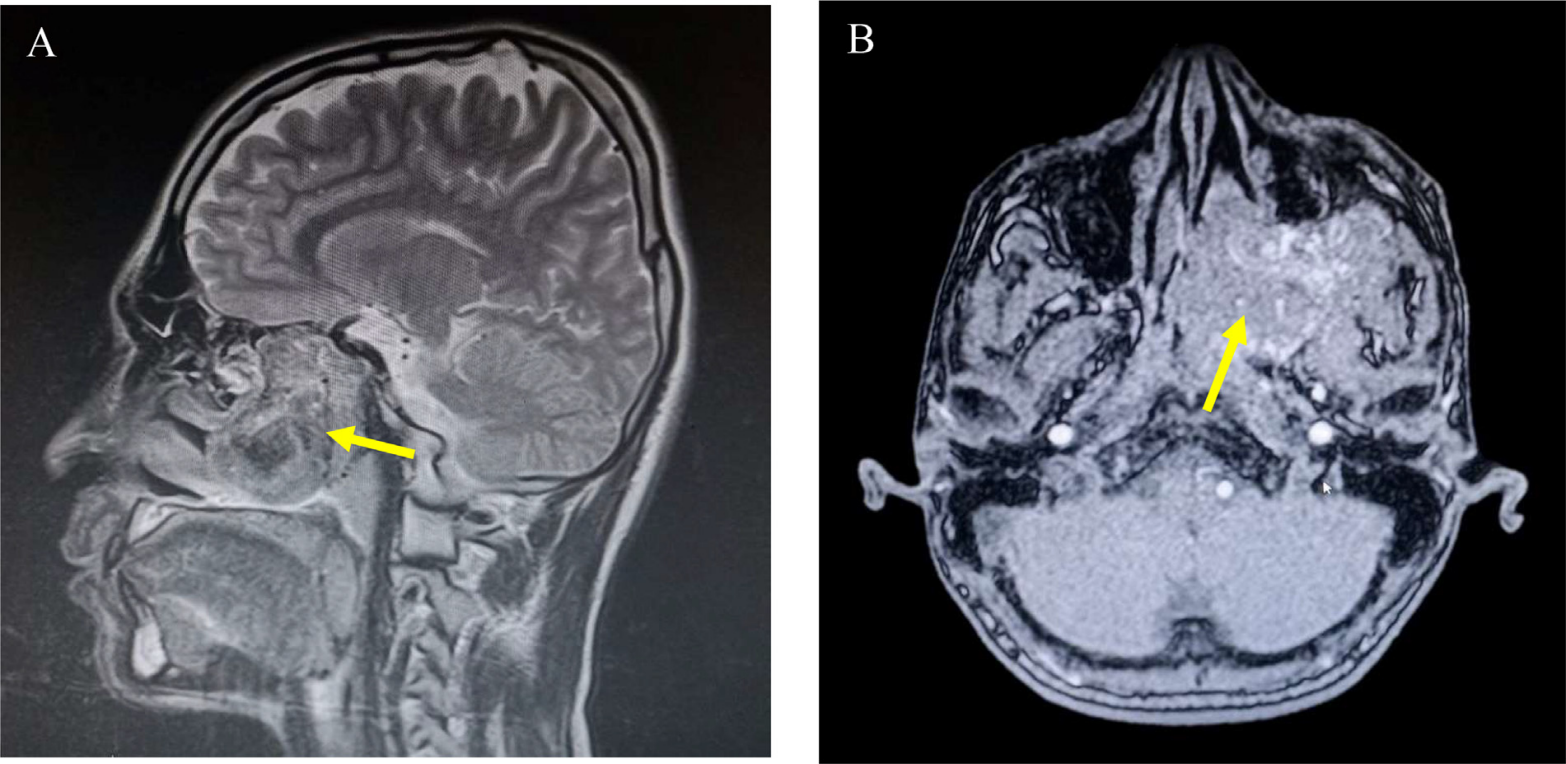
(A) Sagittal T2-weighted image: Demonstrates a large, heterogeneously hyperintense mass occupying the nasopharynx and extending superiorly. The yellow arrow highlights the extensive nature of the lesion. (B) Axial T1-weighted postcontrast image: Shows intense heterogeneous enhancement of the mass. The yellow arrow points to multiple punctate and curvilinear “flow voids” (dark signal spots), which represent high-velocity blood flow within the tumor’s rich vascular network, a characteristic feature of Juvenile Nasopharyngeal Angiofibroma (JNA).

Radiological findings: To accurately evaluate the lesion, two distinct imaging modalities were utilized. Initially, MRI of the brain and nasopharynx was performed, revealing a large, well-defined soft tissue mass centered in the nasopharynx, measuring approximately 72 × 49 mm. On T1-weighted imaging, the mass exhibited an intermediate signal intensity with multiple internal punctate and curvilinear flow voids (Fig. 2), suggesting a highly vascular nature. On T2-weighted sequences, the lesion showed a heterogeneously high signal intensity. The MRI demonstrated significant local aggression, with the mass extending into the orbital apex, explaining the patient’s proptosis, and expanding laterally to widen the pterygopalatine and infratemporal fossae.

**Fig. 3:**
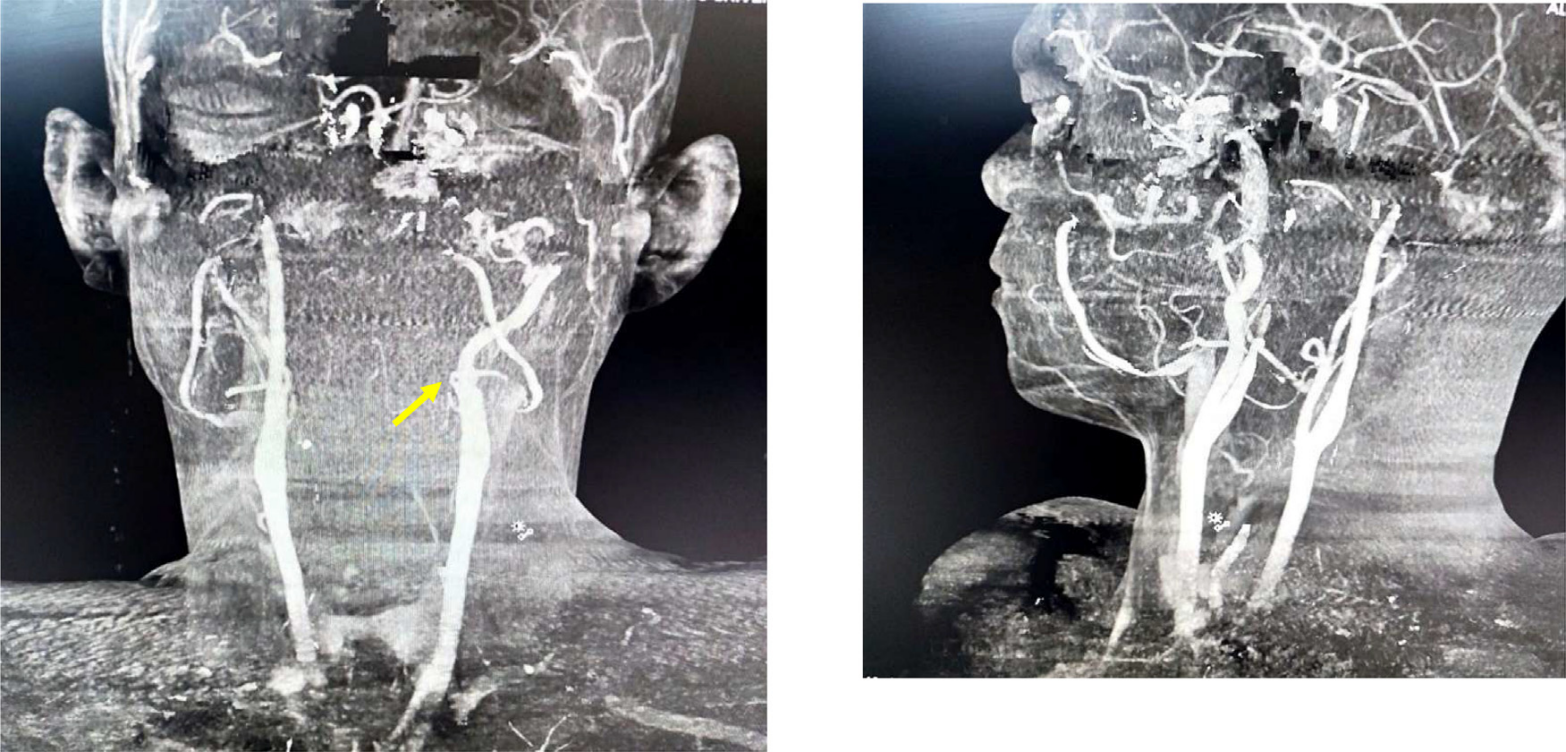
CT angiography, 3D vascular mapping of the head and neck. The yellow arrow highlights the atypical direct arterial supply originating from the left external carotid artery.

Subsequently, CT Angiography with contrast enhancement was performed to delineate the tumor’s extent and vascularity. The study revealed a highly vascular mass receiving an atypical and direct arterial supply originating from the left external carotid artery (ECA) (Fig. 3). The tumor demonstrated significant expansion, extending into the middle cranial fossa through the superior orbital fissure. Furthermore, the mass was found to be compressing the left superior ophthalmic vein, which directly explains the clinical manifestation of proptosis. Additionally, CT scans confirmed the pathognomonic anterior bowing of the posterior wall of the left maxillary sinus, consistent with a positive Holman–Miller sign (Fig. 1).

## Discussion

Atypical clinical presentation and diagnostic delay:

JNA is a benign but locally aggressive tumor that [4] typically manifests with progressive nasal obstruction and recurrent epistaxis.

In this case, the patient presented with unilateral proptosis as the primary and only initial manifestation, which is considered an atypical “ophthalmic-first” presentation.

Orbital involvement generally indicates advanced disease and is reported in only 10%-15% of cases [4].

The history of minor head trauma in our patient led to an initial clinical suspicion of a CCF, highlighting how JNA can mimic traumatic vascular abnormalities when nasal symptoms are absent or neglected.

Unique vascular supply: A comparative analysis

Typically, the primary blood supply for JNA is derived from the Internal Maxillary Artery [4], a branch of the ECA.

However, the CT Angiography in our patient revealed a unique vascular pattern: a direct feeding branch originating from the left ECA.

This direct arterial supply is clinically significant as it increases the risk of massive intraoperative hemorrhage and necessitates precise preoperative embolization.

The highly vascular nature of the lesion was further confirmed by intense contrast enhancement and the presence of “flow voids” on MRI.

The vascular supply in this case is particularly remarkable. While the Maxillary Artery is the classic feeder, a direct supply from the ECA trunk is exceedingly [8,9] rare in the literature.

This atypical vascular mapping complicates the embolization process, as the feeder is more proximal, potentially increasing the risk of reflux into the main ECA during intervention. This unique finding highlights why CT Angiography is indispensable for preoperative mapping in advanced Stage IVb cases [4].

Radiologic diagnosis and local aggression:

The diagnosis was radiologically confirmed by the presence of the Holman–Miller sign [4] (anterior bowing of the posterior wall of the maxillary antrum) (Fig. 1) on CT scans.

The tumor exhibited significant local aggression, extending through the inferior orbital fissure into the orbit and through the superior orbital fissure into the middle cranial fossa.

MRI identified a 72 × 49 mm mass centered in the nasopharynx with intracranial extension.

Based on the invasion of the middle cranial fossa and the resulting compression of the left temporal lobe, the lesion was classified as Stage IVb according to the Fisch classification [5,6] and Stage IIIb according to the Radkowski system.

Multidisciplinary management challenges:

Due to the tumor’s advanced stage (Fisch Stage IVb) and its complex vascularization, specifically the direct supply from the ECA, the case was discussed by a multidisciplinary team. The primary concern was the high risk of uncontrollable intraoperative hemorrhage. Consequently, a neurosurgical and otorhinolaryngology consultation was sought, and the patient was referred to a vascular surgery specialist outside our facility to evaluate the possibility of specialized preoperative embolization or vascular control.

Unfortunately, following this referral, the patient did not return to our department for further management. Due to the patient’s noncompliance and the lack of a centralized tracking system, he was marked as lost to follow-up. No surgical intervention or radiotherapy was performed at our center.

The loss of patient follow-up in this case highlights a significant clinical challenge. To prevent such outcomes, strategies such as enhanced patient education regarding the risks of JNA and establishing a centralized multidisciplinary tracking system are essential to ensure coordination between different specialists and facilities.

## Conclusion

JNA must be considered in the differential diagnosis of any adolescent male presenting with unilateral proptosis, even when classical nasal symptoms are initially absent.

Clinical suspicion of vascular traumatic lesions, such as CCF, following minor head trauma can delay the diagnosis of underlying tumors; therefore, early multiplanar imaging is vital for an accurate diagnosis.

Identifying atypical vascular patterns, such as direct feeding branches from the ECA instead of the typical maxillary artery, is essential for surgical planning and effective hemorrhage control.

Advanced cases (Stage IVb) involving intracranial extension and temporal lobe compression represent a significant surgical challenge that necessitates a multidisciplinary approach, integrating the expertise of both Otorhinolaryngology and Neurosurgery teams.

## Provenance and peer review

Not commissioned, externally peer-reviewed.

## Patient consent

Written informed consent for the publication of this case report was obtained from the patient.

## Appendix Supplementary materials

Image, application 1
