# Supplementary material for: Unilateral proptosis as the primary presentation of Stage IVb Juvenile Nasopharyngeal Angiofibroma with direct external carotid artery supply: Case report
Source: Radiol Case Rep. 2026 Apr 16;21(7):2777–81. doi: 10.1016/j.radcr.2026.03.024 (PMC13099449; doi:10.1016/j.radcr.2026.03.024)
Supplement: Supplementary file 1 [file mmc1.docx]

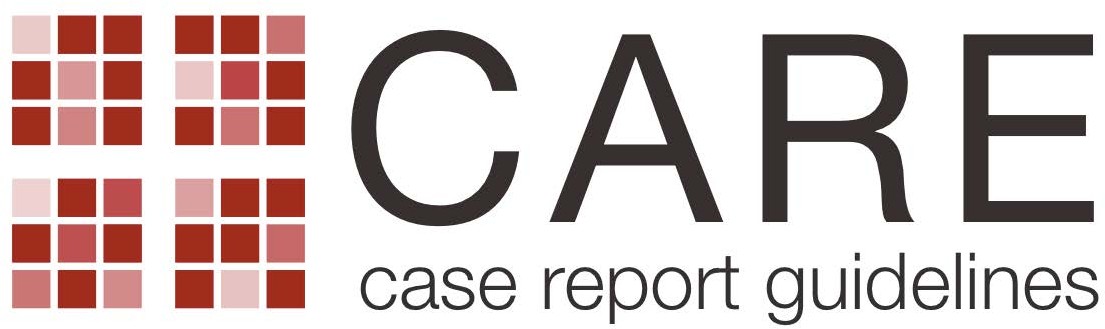
CARE Checklist of information to include when writing a case report
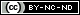


**Topic Item Checklist item description Reported on Page**

**Title 1** The diagnosis or intervention of primary focus followed by the words “case report” page 1

**Key Words 2** 2 to 5 key words that identify diagnoses or interventions in this case report, including "case report" page 2

# Abstract

**(no references)**

**3a** Introduction: What is unique about this case and what does it add to the scientific literature? Page 2

**3b** Main symptoms and/or important clinical findings page 2

**3c** The main diagnoses, therapeutic interventions, and outcomes page 2

**3d** Conclusion—What is the main “take-away” lesson(s) from this case? Page 2

**Introduction 4** One or two paragraphs summarizing why this case is unique (**may include references**) page 3

**Patient Information 5a** De-identified patient specific information page 3

**5b** Primary concerns and symptoms of the patient page 3

**5c** Medical, family, and psycho-social history including relevant genetic information page 3

**5d** Relevant past interventions with outcomes page 3

# Clinical Findings

**Timeline**

**Diagnostic Assessment**

**Therapeutic Intervention**

**Follow-up and Outcomes**

1. Describe significant physical examination (PE) and important clinical findings page 3
2. Historical and current information from this episode of care organized as a timeline page 3

**8a** Diagnostic testing (such as PE, laboratory testing, imaging, surveys). Page 3

**8b** Diagnostic challenges (such as access to testing, financial, or cultural) page 4

**8c** Diagnosis (including other diagnoses considered) page 4

**8d** Prognosis (such as staging in oncology) where applicable N/A

**9a** Types of therapeutic intervention (such as pharmacologic, surgical, preventive, self-care) page 3-4

**9b** Administration of therapeutic intervention (such as dosage, strength, duration) page 3-4

**9c** Changes in therapeutic intervention (with rationale) N/A

**10a** Clinician and patient-assessed outcomes (if available) page 4

**10b** Important follow-up diagnostic and other test results page 5

**10c** Intervention adherence and tolerability (How was this assessed?) N/A

**10d** Adverse and unanticipated events N/A

**Discussion 11a** A scientific discussion of the strengths AND limitations associated with this case report page 4

**11b** Discussion of the relevant medical literature **with references** page 4

**11c** The scientific rationale for any conclusions (including assessment of possible causes) page 5

**11d** The primary “take-away” lessons of this case report (without references) in a one paragraph conclusion page 5

**Patient Perspective 12** The patient should share their perspective in one to two paragraphs on the treatment(s) they received page 5

**Informed Consent 13** Did the patient give informed consent? Please provide if requested .**NO-** Unnecessary, information taken from the patient's file
